# Human Pathogens Abundant in the Bacterial Metagenome of Cigarettes

**DOI:** 10.1289/ehp.0901201

**Published:** 2009-10-22

**Authors:** Amy R. Sapkota, Sibel Berger, Timothy M. Vogel

**Affiliations:** 1 Maryland Institute for Applied Environmental Health, University of Maryland College Park School of Public Health, College Park, Maryland, USA; 2 Environmental Microbial Genomics Group, Laboratoire Ampère, Ecole Centrale de Lyon, France

**Keywords:** bacteria, bacterial metagenome, cigarettes, pathogens, smoking, tobacco

## Abstract

**Background:**

Many studies have evaluated chemical, heavy metal, and other abiotic substances present in cigarettes and their roles in the development of lung cancer and other diseases, yet no studies have comprehensively evaluated bacterial diversity of cigarettes and the possible impacts of these microbes on respiratory illnesses in smokers and exposed nonsmokers.

**Objectives:**

The goal of this study was to explore the bacterial metagenomes of commercially available cigarettes.

**Methods:**

A 16S rRNA-based taxonomic microarray and cloning and sequencing were used to evaluate total bacterial diversity of four brands of cigarettes. Normalized microarray data were compared using principal component analysis and hierarchical cluster analysis to evaluate potential differences in microbial diversity across cigarette brands.

**Results:**

Fifteen different classes of bacteria and a broad range of potentially pathogenic organisms were detected in all cigarette samples. Most notably, we detected *Acinetobacter*, *Bacillus*, *Burkholderia*, *Clostridium*, *Klebsiella, Pseudomonas aeruginosa,* and *Serratia* in ≥ 90% of all cigarette samples. Other pathogenic bacteria detected included *Campylobacter, Enterococcus, Proteus,* and *Staphylococcus.* No significant variability in bacterial diversity was observed across the four different cigarette brands.

**Conclusions:**

Previous studies have shown that smoking is associated with colonization by pathogenic bacteria and an increased risk of lung infections. However, this is the first study to show that cigarettes themselves could be the direct source of exposure to a wide array of potentially pathogenic microbes among smokers and other people exposed to secondhand smoke. The overall public health implications of these findings are unclear at this time, and future studies are necessary to determine whether bacteria in cigarettes could play important roles in the development of both infectious and chronic respiratory diseases.

Cigarette smoking adversely impacts almost every organ system of the human body [International Agency for Research in Cancer (IARC 2004)]. It is a leading risk factor of mortality and morbidity in developed and developing countries (Ezzati and Lopez 2003) and is exceedingly costly to societies worldwide (Centers for Disease Control and Prevention 2002; Yach et al. 2005). Smoking has been well established as the principal cause of lung cancer (IARC 2004) and the leading risk factor for chronic obstructive pulmonary disease (Mannino and Buist 2007; Murin et al. 2000). Moreover, smoking is increasingly being recognized as a risk factor for a wide array of other respiratory illnesses in children and adults, including the common cold, influenza, asthma, bacterial pneumonia, and interstitial lung disease, to name a few (Murin et al. 2000). Over 3,000 chemicals, heavy metals, and other constituents have been isolated from tobacco (IARC 2004), and the overwhelming majority of studies that have investigated adverse health impacts associated with cigarette smoking have focused on the role of these compounds, as well as particulate matter, on pulmonary and systemic pathophysiologic changes that can lead to disease. Few studies however, have investigated bacterial components of cigarettes and their possible roles in smoking-associated illnesses (Bogden et al. 1981; Eaton et al. 1995; Hasday et al. 1999; Kurup et al. 1983; Larsson et al. 2008; Morishita 1983; Pauly et al. 2008; Rooney et al. 2005; Squires and Hayes 1961).

To date, the studies that have been conducted have used traditional culture-based detection methods and have focused on only a limited number of microorganisms such as *Bacillus* spp. (Rooney et al. 2005), *Pantoea* spp. (Larsson et al. 2008), *Kurthia* spp. (Rooney et al. 2005), *Mycobacterium avium* (Eaton et al. 1995), and *Actinomycetes* spp. (Kurup et al. 1983). Thus, very little is known about the prevalence and diversity of microorganisms in cigarettes. Yet, in an era where microbes not only cause acute infectious illnesses but also are increasingly being recognized as etiologic agents or risk factors for chronic diseases including cancers (Correa 2003; Hohenberger and Gretschel 2003; Parsonnet 1995) and neurologic disorders (McKee and Sussman 2005; Schulz et al. 2006), it is perhaps critical that we further our understanding of the bacterial diversity of cigarettes, which are used by over 1.2 billion people (≥ 15 years old) worldwide (IARC 2004).

In this study, we explored the bacterial metagenome of commercially available cigarettes using a 16S rRNA-based taxonomic microarray, as well as traditional cloning and sequencing methods, to better understand bacterial diversity of these widely used products. This is the first study to show that the number of microorganisms in cigarettes may be as vast as the number of chemical constituents in these products.

## Materials and Methods

### Sample collection

In January 2007, cigarettes (*n* = 20 packs) were purchased from five randomly selected tobacco stores in Lyon, France. Four cigarette brands were included: Marlboro Red (Philip Morris, Inc., Richmond, VA, USA), Camel (R.J. Reynolds Tobacco Co., Winston-Salem, NC, USA), Kool Filter Kings (British American Tobacco Group, London, England), and Lucky Strike Original Red (British American Tobacco Group, London, England). These brands are among the most commonly smoked brands of cigarettes in Westernized countries and represent three major tobacco companies. All of the cigarettes were made in the European Union.

### DNA extraction

Cigarette packs were opened in a sterilized biological safety cabinet. Using sterile gloves, five cigarettes from each package were dissected, and the tobacco from all five cigarettes, equaling 3.5 g, was combined in a sterile centrifuge tube. Total metagenomic DNA was extracted from each tobacco sample using the UltraClean Mega Soil DNA Isolation Kit (MoBio Laboratories, Inc., Carlsbad, CA, USA). Resulting DNA was purified using the NucleoSpin Extract 2 Kit (Macherey-Nagel Eurl, Hoerdt, France).

### Polymerase chain reaction, cloning, and sequencing

16S rRNA genes present in purified metagenomic DNA were amplified using universal primers pA and pH′ (Bruce et al. 1992) to obtain 16S amplicons representative of the total bacterial community present in the cigarette samples. The pA primer was amended to include T7 promoter for subsequent labeling. Primer sequences (5′ to 3′) were as follows: pA-T7; TAA TAC GAC TCA CTA TAG AGA GTT TGA TCC TGG CTC AG: pH′; AAG GAG GTG ATC CAG CCG CA. The polymerase chain reaction (PCR) mixture yielded a final solution containing 1X TITANIUM Taq PCR buffer (Clontech Laboratories, Inc., Mountain View, CA, USA), 200 μM deoxynucleotide triphosphates, 0.5 μM of each primer, 1.5 units of TITANIUM Taq, and approximately 150 ng metagenomic DNA. Purified metagenomic soil DNA and molecular-grade water were used as positive and negative controls, respectively. Thermal cycling conditions were as follows: 94°C for 3 min; 35 cycles of 94°C for 45 sec, 55°C for 45 sec, 72°C for 90 sec; and a final extension at 72°C for 5 min. Results were visualized by gel electrophoresis. Gel bands of 16S amplicons (1,500 bp) were extracted using sterilized blades and purified using the GFX PCR DNA and Gel Band Purification Kit (GE Healthcare, Piscataway, NJ, USA). All PCR reactions were prepared in a sterilized biological safety cabinet, and all amplicons were analyzed in a dedicated post-PCR area.

Purified 16S amplicons from one of the Marlboro Red metagenomic samples were cloned for sequencing using a TOPO TA cloning kit (Invitrogen, Cergy Pontoise, France) with a pCR4 TOPO vector and One Shot TOP10 electrocompetent cells (Invitrogen). Resulting colonies (*n* = 288) were isolated and analyzed to check for positive clones and to choose clones with different restriction profiles. Each colony was inoculated in 100 μL LB containing 100 μg/mL ampicillin and grown overnight at 37°C. One microliter of each overnight culture was used for a subsequent PCR reaction. Amplification was performed with the universal M13 reverse and M13 forward primer pairs using a Platinum PCR SuperMix 96 (Invitrogen).

Each amplification product was digested by *Eco*RI, and restriction profiles were observed by gel electrophoresis. Positive clones (*n* = 96) were selected and sequenced with the M13 primers by the ABI3730x/DNA Analyzer system (Cogenics, Meylan, France). The DNA sequences were analyzed by Lasergene 7.2 software (DNAStar Inc., Madison, WI, USA). For identification of closest relatives, the consensus sequences were compared with 16S rRNA gene sequences in GenBank databases using the NCBI Blast search tool (National Center for Biotechnology Information 2009).

### Labeling and sample preparation

Purified 16S amplicons from all samples were reverse-transcribed and labeled with UTP-Cy3. The reaction mixture yielded a final solution of 1X T7 RNA buffer; 10 mM DTT; 0.5 mM concentrations of dATP, dCTP, dGTP, and dUTP; 20 units of RNasin (Invitrogen), 1 μL T7 RNA polymerase (Invitrogen), 0.25 mM UTP-Cy3, and 400 ng purified PCR amplicons. Each reaction mixture was incubated in the dark at 37°C for 4 hr. Resulting RNA was purified using the RNeasy Mini Kit (Qiagen, Valencia, CA, USA). Purified RNA was then quantified, and frequency of incorporation (FOI) of Cy3 was calculated: FOI of Cy3 = (OD550/0.15)*(324)/OD260*40). Purified RNA was then fragmented in a reaction mixture yielding a final solution of 25 μM Tris Cl and 10 mM ZnSO_4_. The fragmentation reactions were incubated for 30 min at 60°C, and 1.43 μL 500 mM EDTA was added to stop each reaction.

### Microarray hybridization and scanning

The 16S rRNA-based taxonomic microarray slides (Schott Nexterion AG, Mainz, Germany) and probes (positive controls and targets) (Eurogentec, Seraing, Belgium) were custom designed and synthesized as previously described (Sanguin et al. 2006a, 2006b). Briefly, the microarray included 742 unique 20mer probes that targeted a broad array of bacterial phyla, classes, orders, families, genera, and species, and two positive control probes that targeted the Eubacteriaceae. The probe pattern on the microarray included two spots of each of the positive control probes and one spot of each of the other probes; this pattern was repeated six times on each microarray slide. Based on a comparison of experimental and theoretical hybridizations, the false-positive and false-negative rates of this microarray system have been calculated as 0.91% and 0.81%, respectively (Sanguin et al. 2006b).

Labeled and fragmented RNA was prepared in a hybridization mixture yielding a final solution of 0.1% SDS, 1X Denhardt’s solution (Sigma Chemical Company, St. Louis, MO, USA), 6X SSC, and 300 ng RNA. Slides were prehybridized, hybridized, and washed in an A-Hyb Hybridization Station (Miltenyi Biotec GmbH, Bergisch Gladbach, Germany). Prehybridization was performed at 57°C for 10 min, hybridization was performed at 57°C for 240 min, and a four-step wash cycle was performed at 20°C for 4 min. Slides were dried by centrifugation in a microcentrifuge for 2 min at top speed. Scanning was performed using a GenePix Personal 4100A scanner (Molecular Devices Corporation, Sunnyvale, CA, USA), and data were analyzed using GenePix Pro 6 Microarray Image Analysis (Molecular Devices Corporation).

### Data filtration, normalization, and analysis

Filtration, normalization, and data analysis were performed using the R Project for Statistical Computing (http://www.r-project.org/). Data filtration and normalization were performed as described by Sanguin et al. (2006a). Normalized microarray results for the cigarette samples were compared by principal component analysis (PCA) and hierarchical cluster analysis using the ade4 package (http://pbil.univ-lyon1.fr/ADE-4/).

## Results

**Figure 1:**
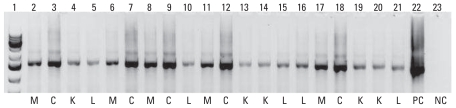
16S PCR amplicons (1,500 bp) generated from metagenomic DNA extracted from cigarettes. Abbreviations: M, Marlboro Red samples; C, Camel samples; K, Kool Filter King samples; L, Lucky Strike Original Red samples; PC, positive control metagenomic DNA sample extracted from soil; NC, negative control. Lane 1, DNA ladder.

All cigarette samples were positive for 16S bacterial rRNA (Figure 1) and without exception, 16S rRNA originating from all samples, regardless of brand, hybridized with over 100 unique microarray probes. Fifteen different classes of bacteria were detected in the cigarette samples (Table 1). Members of the following phyla were detected in nearly all of the samples: *Actinobacteria, Bacteroidetes, Chloroflexi, Cyanobacteria, Firmicutes,* and *Proteobacteria* (*Alphaproteobacteria, Betaproteobacteria, Deltaproteobacteria,* and *Gammaproteobacteria*). As expected, *Nicotiana tabacum* 16S chloroplast rRNA genes also were detected in every sample.

Our microarray was designed to detect microorganisms at both the genus and species level. A variety of environmental bacterial organisms were identified in all samples, including *Amaracoccus, Legionellales, Methylobacterium, Nostoc, Paracoccus*, *Pseudomonas chlororaphis,* and *Pseudomonas cichorii*, to name a few. A broad range of gram-positive and gram-negative bacterial genera and species medically important to humans and/or potential human pathogens were also detected in the cigarette samples (Table 2). Most notably, the following organisms were detected in ≥ 90% of all samples: *Acinetobacter, Bacillus, Burkholderia, Clostridium, Klebsiella oxytoca, Pseudomonas*, including *Pseudomonas aeruginosa* and *Pseudomonas stutzeri,* and *Serratia* sp.

As anticipated (DeSantis et al. 2007), the cloning and sequencing approach detected some but not all of the bacterial organisms detected with the microarray (Tables 1 and 2). Only five bacterial classes were identified with cloning and sequencing compared with 15 classes identified with the microarray (Table 1). In addition, only 27 unique bacterial genus or species were identified with cloning and sequencing. Two of these organisms, *Bacillus* spp. and *Pseudomonas* spp., were also detected in cigarettes using the microarray approach (Table 2). Other organisms detected by cloning and sequencing, but not by the microarray, included *Aurantimonas altamirensis, Enterococcus gallinarum,* and *Staphylococcus* spp. (Table 2), which were not represented by probes present on the microarray.

**Figure 2:**
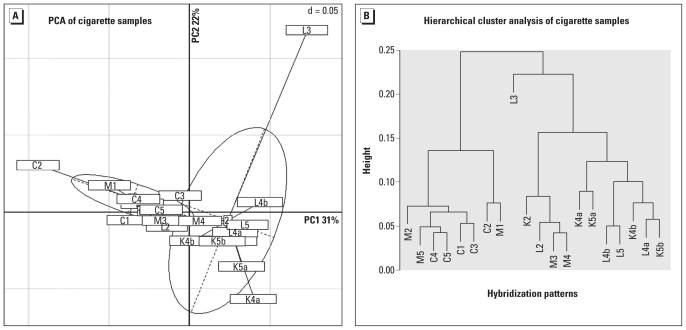
Hybridization pattern analysis of bacterial diversity in four brands of cigarettes performed by PCA (*A*) and hierarchical cluster analysis (*B*). In Panels *A* and *B*, the letters within the sample codes represent the following cigarette brands: M, Marlboro Red; C, Camel; K, Kool Filter Kings; and L, Lucky Strike Original Red.

**Figure 3:**
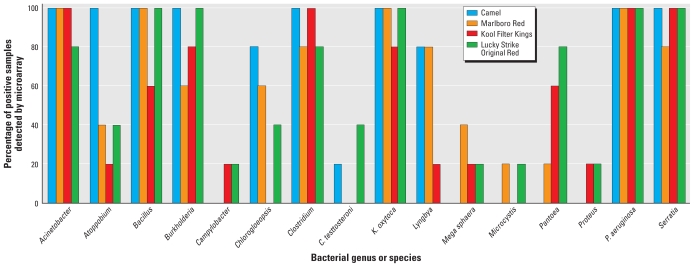
Distribution of select bacteria of importance to human health detected in different cigarette brands using a 16S rRNA-based taxonomic microarray.

The PCA showed that samples originating from the four different cigarette brands were not well separated along the first or second PCA axes (Figure 2A). In other words, variability in bacterial diversity between cigarette brands was not great, except for a few outlying samples (Figures 2A, 3). Nevertheless, the hierarchical cluster analysis showed that the Marlboro Red and Camel cigarette samples tended to cluster together, whereas the Kool Filter Kings and Lucky Strike Original Red cigarette samples were similar to one another (Figure 2, Panel B). Microarray probes targeting *Pseudomonas* clusters, some *Gammaproteobacteria,* and some *Betaproteobacteria* contributed significantly to the hybridization patterns observed for the Kool Filter Kings and Lucky Strike Original Red cigarettes, which distinguished them from the Marlboro Red and Camel samples.

## Discussion

We explored the bacterial metagenome of commercially available cigarettes and revealed for the first time that these widely used products are characterized by a broad array of bacterial diversity. Regardless of brand, tested cigarettes harbored numerous gram-positive and gram-negative bacterial types, ranging from soil microorganisms and commensals to potential human pathogens, including *Acinetobacter*, *Bacillus, Burkholderia, Clostridium*, *Klebsiella*, and *Pseudomonas aeruginosa* (Table 2). Many of the detected organisms are capable of causing pneumonia, bacteremias, foodborne illnesses, meningitis, endocarditis, and urinary tract infections, to name a few. For example, *P. aeruginosa*—a bacterium detected in 100% of all cigarette samples tested in this study—alone causes 10% of all hospital-acquired infections in the United States and is the leading cause of nosocomial pneumonia in both Europe and the United States (Bergogne-Berezin 1995).

The identification of *Bacillus* spp. by both our microarray approach and cloning and sequencing is consistent with previous culture-based work by both Rooney et al. (2005) and Larsson et al. (2008). Rooney et al. (2005) identified eight species of *Bacillus* in cigarettes collected from military personnel during an investigation of acute eosinophilic pneumonitis among individuals who had been deployed during Operation Iraqi Freedom. Larsson et al. (2008) recovered *Bacillus* spp., including *Bacillus subtilis,* from fresh tobacco leaves collected at a tobacco-manufacturing plant. *Bacillus* spp. have also been identified in cured tobacco leaves (Kaelin and Gadani 2000), stored tobacco (Kaelin et al. 1994), dead tobacco beetles (Kaelin et al. 1994), and chewing tobacco sold in the United States (Rubinstein and Pedersen 2002).

In addition to *Bacillus* spp., a few of the other species detected by our microarray approach including *Pantoea* spp., *Acinetobacter* spp., *Pseudomonadaceae* spp., and members of the Enterobacteriaceae family were recently detected in a study by Larsson et al. (2008) that investigated microbiological components of fresh tobacco leaves using culture methods. These investigators identified the following bacterial species in fresh tobacco leaves using blood agar, eosin methylene blue agar, and half-strength tryptic soy agar: *Pantoea agglomerans, Acinetobacter calcoaceticus,* and specific Pseudomonadaceae species such as *P. fluorescens* and *Stenotrophomonas maltophilia.* As outlined in Table 2, we also detected *Pantoea* spp., *Acinetobacter* spp. and *Stenotrophomonas maltophilia* in cigarette tobacco, along with other specific Pseudomonadaceae species including *P. aeruginosa* and *P. stutzeri.* Furthermore, Larsson et al. (2008) detected some members of the Enterobacteriaceae family such as *E. amnigenus* and *E. cancerogenus.* Although we did not detect these organisms, because probes for these species were not included in our microarray, we did detect other members of this gram-negative family including *Klebsiella oxytoca* and *E. coli.*

The findings that Larsson et al. (2008) were able to culture a few of the same organisms from fresh tobacco leaves that we detected in commercially available cigarettes provides evidence that cigarette tobacco may be contaminated with bacteria early in the production process, possibly at the farm level, and that the organisms are likely able to survive the manufacturing process, including the curing process, and remain present in consumer-ready cigarettes. Moreover, the fact that Larsson et al. (2008) used fairly nonspecific culture media and were subsequently able to identify some of the same organisms that we detected provides intriguing support for the idea that many other organisms that we detected via microarray also could be cultured if more selective media are used in future culture-based studies of cigarettes.

One other previous study cultured bacteria from single tobacco flakes recovered from commercially available cigarettes, as well as fine tobacco dust that potentially could be inhaled into deeper regions of the lungs (Pauly et al. 2008). These researchers observed that 92.9% of tobacco flakes representing eight different cigarette brands were positive for bacterial growth after 24 hr. In addition, 90% of tobacco dust samples tested positive for bacterial growth. Although these researchers did not identify the specific bacterial species that were isolated, their findings provide additional evidence that cigarettes are widely contaminated with bacteria and that the organisms are viable.

Important questions remain, however, regarding the implications of bacteria-harboring cigarettes. Can bacteria present in cigarettes survive the burning/smoking process, be inhaled by smokers and other exposed individuals, and colonize the lungs? In a study by Eaton et al. (1995), the authors recovered *Mycobacterium avium* from smoked cigarette filters, providing evidence that these microorganisms can survive in the presence of high temperatures and gases generated by a lit cigarette (Eaton et al. 1995). Thus, it is possible that other organisms, particularly the hardy endospore formers including *Bacillus* spp. and *Clostridium* spp. identified in the present study, also could survive the harsh conditions of the cigarette burning/smoking process. However, beyond the Eaton et al. (1995) study, no researchers to our knowledge have investigated the survival of other bacterial species in smoked cigarettes. This is a very important future avenue of research that needs to be investigated to fully understand the potential public health implications of bacterial pathogens present in cigarettes. On the other hand, Pauly et al. (2008) demonstrated that bacteria can even be cultured from fine tobacco powder present in commercially available cigarettes. Therefore, it is possible that bacteria associated with these fine particles could pass through cigarette filters currently used (Pauly et al. 2002) and be inhaled into the lungs even after the first few puffs of a cigarette, when the majority of the cigarette remains at low temperatures. Moreover, because tobacco flakes and particles are often observed on the tips of cigarette filters (Pauly et al. 2008) and subsequently brought into the mouth, it is possible that bacterial organisms present in the cigarette could be transferred to the mouths of smokers even before the cigarette is lit.

In terms of the cigarette smoke itself, the microbiology of this complex mixture has not been studied comprehensively. To date, most studies concerning microbiological components of cigarette smoke have focused on endotoxins (Barnes and Glantz 2007; Hasday et al. 1999; Larsson et al. 2004; Reiman and Uitti 2000; Rennie et al. 2008; Sebastian et al. 2006; Thorne et al. 2009). Hasday et al. (1999) demonstrated for the first time that both mainstream and sidestream cigarette smoke contain significant levels of bioactive bacterial endotoxin (ranging from 18 ± 1.5 ng/cigarette to 120 ± 64 ng/cigarette). Since then, other groups have shown that smoking indoors significantly increases indoor air endotoxin concentrations in experimental settings (Larsson et al. 2004), as well as in homes (Rennie et al. 2008; Sebastian et al. 2006). In addition to endotoxins, Larsson et al. (2008) recently showed that elevated levels of muramic acid, a peptidoglycan marker that can serve as an indicator of gram-positive bacteria, are also present in tobacco smoke. However, beyond these bacterial markers, very little work has been performed to evaluate whole, viable, particle-associated bacterial cells that may be aerosolized in tobacco smoke, and this is certainly an exciting potential avenue for future research.

If viable bacterial cells are ultimately detected in tobacco smoke, then they would have to subsequently colonize exposed individuals to cause health effects. Although data on the bacterial diversity of tobacco smoke are not available, previous studies have shown that active smoking and exposure to secondhand smoke are associated with colonization by potentially pathogenic bacteria (Brook and Gober 2005, 2008; Shiloah et al. 2000) and an increased risk of acute bacterial lung infections (Aronson et al. 1982). The nasopharyngeal and oral flora of smokers and children of smokers is characterized by more bacterial pathogens compared with that of nonsmokers and their children (Brook and Gober 2005, 2008). In fact, smokers are 18 times more likely to harbor bacterial pathogens in the oral cavity compared with nonsmokers (Shiloah et al. 2000). In addition, the elevated number of pathogens in the nasopharyngeal cavities of smokers has been shown to revert to normal levels observed in nonsmokers after complete smoking cessation (Brook and Gober 2007).

Our findings provide intriguing evidence that the source of pathogenic organisms in smokers (and those impacted by secondhand smoke) may be the cigarettes themselves. However, it is important to note that some bacterial organisms recovered from smokers commonly colonize the nasopharynx, and increased susceptibility to colonization, as well as the increased risk of lung infections associated with smoking, could be due to immunosuppressive activities by chemicals and particulate matter present in cigarette smoke. Long-term smokers may have less-effective mucociliary clearance mechanisms (Wanner et al. 1996), chronic inflammation of the lungs (Yanbaeva et al. 2007), and compromised host defense mechanisms (Birrell et al. 2008), all of which could contribute to higher levels of colonization. Future work is necessary to determine possible interactions or synergisms between colonization that may result because of immunosuppressive effects and colonization that may occur as a result of the introduction of organisms from external sources.

Beyond the issue of bacterial colonization and acute respiratory illnesses, another avenue to explore is whether bacteria in cigarettes could also contribute to human carcinogenesis. The role of microbes in the development of some human cancers is being explored (Correa 2003; Hohenberger and Gretschel 2003; Parsonnet 1995). Although the specific microbes identified as the causative agents in these cancers have not been detected in tobacco products, further studies are needed to understand whether bacteria originating from cigarettes could contribute to carcinogenesis either through biologically mediated pathways or through biological and chemical interactions. Recently, Hunter et al. (2005) showed that *Bacillus subtilis*, which was detected in 90% of our cigarette samples, is a possible degrader of pyrene and benzo[*a*]pyrene, major chemical carcinogens found in cigarettes (Hunter et al. 2005). Although it is unclear at this time whether the metabolites resulting from the degradation of these compounds by *B. subtilis* are more toxic than their parent compounds (Hunter et al. 2005), it is worthwhile to note that bacteria—including *Bacillus* and others that may be deposited in the lungs as a result of cigarette smoking—could alter cigarette-associated chemicals into greater or lesser carcinogens.

One limitation to this study is that the microarray platform used was primarily developed to explore bacterial diversity in environmental samples. Therefore, the probes present on the microarray slides more heavily represented soil and water microorganisms versus potential human pathogens, such as those included in the Enterobacteriaceae family. The implication of this limitation is that there could be even more potential bacterial pathogens present in the cigarette samples compared with the number that we detected with our current microarray system.

Another limitation of this study is that we analyzed cigarette tobacco alone and did not include analyses of cigarette smoke. Future studies are necessary to fully characterize the microbiology of cigarette smoke. Beyond the smoke, additional studies will be necessary to determine whether organisms present in the cigarettes and smoke can colonize smokers and other exposed individuals and ultimately cause human illnesses.

## Conclusions

Nearly every scientific paper concerning the acute human health effects associated with cigarette smoking mentions that smoking is associated with increases in bacterial infections among smokers. However, to our knowledge no one has comprehensively evaluated whether the actual cigarettes themselves may be the source of exposure to bacterial organisms that subsequently cause infection and other potential illnesses. Here we describe that multiple brands of cigarettes are rich in bacterial diversity, harboring a broad array of microorganisms from environmental bacteria and commensals to potential human pathogens. The overall public health implications of these findings are unclear at this time, and future studies are necessary to determine whether microbes originating from cigarettes could play a considerable role in the development of both infectious and chronic diseases among smokers and other exposed populations.

## Figures and Tables

**Table 1:** Bacterial phyla and classes detected in commonly smoked cigarettes.

Phylum, class	Detected by microarray	Detected by cloning and sequencing
*Actinobacteria, Actinobacteria*	Yes	Yes
*Bacteroidetes, Bacteroidetes*	Yes	No
*Bacteroidetes, Sphingobacteria*	Yes	Yes
*Chloroflexi, Chloroflexi*	Yes	No
*Cyanobacteria, Cyanobacteria*	Yes	No
*Cyanobacteria, Cyanophyceae*	Yes	No
*Deinococcus-Thermus, Thermus*	Yes	No
*Firmicutes, Bacilli*	Yes	Yes
*Firmicutes, Clostridia*	Yes	No
*Planctomycetes, Planctomycetacia*	Yes	No
*Proteobacteria, Alphaproteobacteria*	Yes	Yes
*Proteobacteria, Betaproteobacteria*	Yes	No
*Proteobacteria, Deltaproteobacteria*	Yes	No
*Proteobacteria, Gammaproteobacteria*	Yes	Yes
*Proteobacteria, Epsilonproteobacteria*	Yes	No

**Table 2:** Select bacterial genera and species detected in commonly smoked cigarettes that are medically important to humans.

Genus	Species	Detected by microarray (% of samples)	Detected by cloning and sequencing (% of clones)	Potential human health effects	Previously detected in cigarettes
*Acinetobacter*^a^		Yes (95)	No	Wide range of illnesses,^b^ from pneumonia to bacteremias	No
*Atopobium*^a^		Yes (50)	No	Isolated from a range of infections: periodontal and pelvic abscesses, abdominal wounds	No
*Aurantimonas*	*A. altamirensis*	No	Yes (1)	Isolated from a dendritic corneal ulcer	No
*Bacillus*^a^		Yes (90)	Yes (13)	Individual species can cause a range of illnesses from foodborne illnesses to anthrax	Yes
	*B. pumilus*	No	Yes (8)	Isolated from a central venous catheter infection	Yes
*Burkholderia*^a^		Yes (90)	No	Some species can cause pneumonias and bacteremias	No
*Campylobacter*^a^		Yes (10)	No	Etiologic agent of Campylobacteriosis and Guillain-Barre Syndrome	No
*Chlorogloeopsis*^a^		Yes (45)	No	Type of blue-green algae; potential source of cyanotoxins	No
*Clostridium*^a^		Yes (90)	No	Genus includes human pathogens that can cause a wide range of illnesses: foodborne illnesses, pneumonias, and bacteremias	No
*Comamonas*	*C. testosteroni*	Yes (15)	No	Rarely isolated from a range of infections: meningitis, bacteremias, endocarditis	No
*Corynebacterium*	*C. xerosis*	Yes (10)	No	Pneumonia, bacteremias, and skin infections^b^	No
*Dialister*^a^		Yes (5)	No	Certain species isolated from bacteremias and periodontal disease	No
*Enterococcus*	*E. gallinarum*	No	Yes (1)	Bacteremias, endocarditis, meningitis	No
*Escherichia*	*E. coli K12*	No	Yes (1)	Commonly used research model in the laboratory; other *E. coli* strains vary from harmless to highly pathogenic	No
*Klebsiella*	*K. oxytoca*	Yes (95)	No	Pneumonia, neonatal bacteremias, urinary tract infections, abscesses	No
*Lyngbya*^a^		Yes (45)	No	A genus of cyanobacteria that causes swimmer’s itch	No
*Megasphaera*^a^		Yes (20)	No	Anaerobic bacteria isolated from tonsilloliths and bacterial vaginosis infections	No
*Microcystis*^a^		Yes (10)	No	Type of blue-green algae; potential source of cyanotoxins	No
*Novosphingobium*	*N. aromaticivorans*	No	Yes (1)	May trigger primary biliary cirrhosis	No
*Pantoea*^a^		Yes (40)	No	Some species can cause bacteremias, endocarditis, and wound infections	No
*Proteus*^a^		Yes (30)	No	Some species can cause urinary tract infections, bacteremias, pneumonias, and wound infections	No
*Pseudomonas*^a^		Yes (100)	Yes (3)	Some species are human pathogens	No
	*P. aeruginosa/P. stutzeri clusters*	Yes (100)	No	Opportunistic human pathogens that can cause pneumonia, urinary tract infections, and bacteremias	No
*Serratia*^a^		Yes (95)	No	Opportunistic human pathogens that can colonize respiratory and urinary tracts	No
*Staphylococcus*	*S. saprophyticus*	No	Yes (6)	Urinary tract infections	No
	*S. epidermidis*	No	Yes (1)	Nosocomial pathogen associated with biofilms and foreign bodies	No
	*S. cohnii*	No	Yes (2)	Bacteremias, brain abscesses	No
	*S. sciuri*	No	Yes (1)	Urinary tract infections, endocarditis, wound infections	No
*Stenotrophomonas*	*S. maltophilia*	No	Yes (2)	Pneumonia, urinary tract infections, bacteremias	No

a Probe targeted only the genus level.

b Particularly among immunocompromised individuals.
